# Inkjet Printing of Polypyrrole Electroconductive Layers Based on Direct Inks Freezing and Their Use in Textile Solid-State Supercapacitors

**DOI:** 10.3390/ma14133577

**Published:** 2021-06-26

**Authors:** Zbigniew Stempien, Mohmmad Khalid, Marcin Kozanecki, Paulina Filipczak, Angelika Wrzesińska, Ewa Korzeniewska, Elżbieta Sąsiadek

**Affiliations:** 1 Institute of Textile Architecture, Lodz University of Technology, 90-924 Lodz, Poland; 2 Institute of Chemistry of São Carlos, University of São Paulo, São Carlos 13566-590, Brazil; mkansarister@gmail.com; 3 Department of Molecular Physics, Lodz University of Technology, 90-924 Lodz, Poland; marcin.kozanecki@p.lodz.pl (M.K.); paulina.filipczak@p.lodz.pl (P.F.); angelika.wrzesinska@dokt.p.lodz.pl (A.W.); 4 Institute of Electrical Engineering Systems, Lodz University of Technology, 90-924 Lodz, Poland; ewa.korzeniewska@p.lodz.pl; 5 Department of Mechanical Engineering, Informatics and Chemistry of Polymer Materials, Lodz University of Technology, 90-924 Lodz, Poland; elzbieta.sasiadek@p.lodz.pl

**Keywords:** reactive printing, direct ink freezing, polypyrrole, textile substrates, textile supercapacitor

## Abstract

In this work, we propose a novel method for the preparation of polypyrrole (PPy) layers on textile fabrics using a reactive inkjet printing technique with direct freezing of inks under varying temperature up to −16 °C. It was found that the surface resistance of PPy layers on polypropylene (PP) fabric, used as a standard support, linearly decreased from 6335 Ω/sq. to 792 Ω/sq. with the decrease of polymerization temperature from 23 °C to 0 °C. The lowest surface resistance (584 Ω/sq.) of PPy layer was obtained at −12 °C. The spectroscopic studies showed that the degree of the PPy oxidation as well as its conformation is practically independent of the polymerization temperature. Thus, observed tendences in electrical conductivity were assigned to change in PPy layer morphology, as it is significantly influenced by the reaction temperature: the lower the polymerization temperature the smoother the surface of PPy layer. The as-coated PPy layers on PP textile substrates were further assembled as the electrodes in symmetric all-solid-state supercapacitor devices to access their electrochemical performance. The electrochemical results demonstrate that the symmetric supercapacitor device made with the PPy prepared at −12 °C, showed the highest specific capacitance of 72.3 F/g at a current density of 0.6 A/g, and delivers an energy density of 6.12 Wh/kg with a corresponding power density of 139 W/kg.

## 1. Introduction

Conducting polymers (e.g., polyaniline, polypyrrole, polythiophene, poly(3,4-ethylenedioxythiophene) have attracted much attention in variety of potential applications [1,2,3,4]. Polypyrrole is of a particular interest, mainly because of its easy preparation, tunable doping/dedoping characteristic, and reasonably high electric conductivity [5,6,7,8]. Additionally, it has been successfully employed in various fields, such as supercapacitors [9,10,11,12,13], photoelectric devices [14], power generation [15,16], gas sensors [17,18,19,20], and electronic circuits [21,22]. For conventional conducting polymers, the main disadvantage is their poor mechanical properties, due to the rigidity of the polymer chain backbone, which limits their processability. Thus, it is very difficult to fabricate conducting polymers into the desired shape and structure. In this regard, supports play a crucial role in construction of conducting polymer-based materials for devices’ applications [23,24,25]. However, among them, PPy shows moderate solubility in some polar aprotic organic solvents, which provides relatively easy method for processing PPy, such as electro-spinning [26,27], spin-coating [28,29], inkjet printing [30,31], and electrodeposition [32,33]. To date, it is still challenging to fabricate PPy in form of well-defined and high conductive layers, especially when it is deposited on textile substrates for textronics products. In the field of smart textiles, PPy layers have shown impressive results for example in textile supercapacitors [34,35,36,37,38] where the specific capacitances ranging from 103 F/g to 325 F/g were reported, such as textile actuators [39,40]; textile heaters [41,42,43]; textile gas sensors [44], where the sensing of PPy finished textiles to NH_3_ and HCl gases was indicated; and textile strain sensors [45,46,47], where the possibility of measure the strain in the range of 20–50% was reported. In case of PPy based textile strain sensors, the possibility of measure the strain in the range of 20–50% means, that the measured values of PPy layer resistance are repeatable for the maximum strain, which can be from 20 to 50% dependence on reported solution.

It is well known that the morphology and conductive properties of the resultant PPy layers depend on a number of parameters, such as the type of oxidant, pyrrole/oxidant molar ratio, type and concentration of a dopant, as well as type of counter-ion and solvent and their concentrations in electrochemical methods. Studies have been also conducted on the effect of synthesis or preparation temperature on the conductivity of PPy layers fabricated by monomer oxidation [48] and electrochemical method [49]. Generally, the conductivity of PPy increases as the preparation temperature decreases [48,49]. The PPy synthesized at higher temperatures (room temperature or higher) contains high concentrations of defect sites [48], which are introduced as a result of unwanted side reactions. These reactions are essentially occurring in competition with the desired reaction, α-α coupling of oxidized monomer units to produce a defect-free polymer chain. At low temperatures, close to 0 °C, the rates of these unwanted reactions become much slower relative to the rate of the desired reaction [48]. In the case of electrodeposition method, the roughness and the conductivity of PPy films are related to the change in chain bonding through α–α to β–β [49]. The α–α chain bonding results in a very smooth surface and higher conductivity of the film but if the β–β bonding dominates, the surface of PPy film becomes rough resulting in lower conductivity. Increasing the temperature causes increase in monomer–monomer and monomer–polymer interactions. Hence, this increases the chances of polymerization through α–β and β–β rather than α–α chain bonding [49]. Since the conductivity of PPy increases as the preparation temperature decreases it should be noted that the fabrication of PPy layers on a specified substrate at the temperature below 0 °C has not been reported in literature yet. It can be expected that the decreasing of synthesis temperature below 0 °C should lead to the further limit a defect sites and increase the PPy layer conductivity. A method of fabrication of a highly conductive free standing polypyrrole films prepared by freezing interfacial polymerization has been communicated by Qi et al. [50]. In this method a solution of pyrrole dissolved in a cyclohexane and an aqueous solution containing both oxidant and dopant are separated after mixing due to the immiscibility of water and cyclohexane and kept in a freezer at −20 °C up to complete the polymerization of pyrrole. The conductivity of the as-prepared PPy film was up to 2000 S/cm, which is much higher than that of PPy prepared by other well-known methods. The most disadvantage of the method proposed by Qi et al. [50] is fact that it may be used only for preparation of free standing PPy films, and it cannot be used in deposition of PPy film on specified substrates.

Among the different methods of PPy deposition, the inkjet printing technique is a very attractive as it allows not only for highly reproducible high-resolution patterning but also for preparation of layer-by-layer structures. Inkjet printing is a versatile method for controlled deposition of functional materials with suitable geometry on various substrates [51]. It does not require any contact between the deposition system and the substrate. The only constraint of this technique is the requirement of fluids (inks) with suitable viscosity and surface tension. The most common printing methods such as screen, rotary, and inkjet printing used to manufacture conducting polymers including PPy have been reviewed in detail by Weng et al. [52]. Among different printing methods, inkjet printing techniques are regarded as the most promising mechanisms for producing plastic electronics and textronic systems. They need inks which provide adherent, compact features with appropriate resolution and conductivity. In the case of polymer deposition, ink should contain dissolved or finely dispersible nanoparticles of conducting polymer. The disadvantages of these techniques are clogging of the nozzles of the printing head resulting in a low conductivity of the printing object. The reactive inkjet printing is a method of PPy deposition on different substrates which is free from the disadvantages mentioned above [31]. In this technique, the printheads are filled with water or alcohol solution of low-molecular-weight reactants and thus the clogging of the nozzles is practically fully eliminated. The PPy-printed conductive layers can be obtained by in situ chemical oxidative polymerization of pyrrole by using proper oxidant directly onto the specified substrate. During printing, the electroconductive layer is deposited line by line onto the surface of substrate in such a way that the first nozzle sprayed the selected line of pattern using an aqueous solution of pyrrole and then the second nozzle sprayed the same line of pattern using an aqueous solution of oxidant, e.g., ammonium peroxydisulfate.

Recently, the extrusion-based 3D printing with freeze-casting of aqueous based inks has been reported as a promising technique to form the layers and 3D architectures from different materials, where the prints were solidified during printing by keeping the solidification temperature between −40 °C to 0 °C. This technique was successfully applied to form a 3D architecture made of graphene aerogels [53], highly porous 3D LiFePO_4_ electrodes [54], 3D aerogel architectures inspired by plant cell wall components and structures [55].

Considering the dynamic development of textronics, textile supercapacitors deserve special attention. They are considered to use in the near future as a flexible power system for electronic circuits in textronic applications to the counterpart of rigid batteries that reduce the comfort of wearable textronic garments. However, having fast charging and high-power abilities, the current challenge is to improve their energy density. Basically, two approaches are considered in the design of textile supercapacitors. In the first approach, material electrodes are placed on two threads, which are then twisted and soaked with electrolyte [38,56,57]. In the second approach (the most commonly used) the material electrodes are applied to the surfaces of two fabrics and then joined with a solid electrolyte through a separator [34,35,37,38,58,59,60,61,62,63,64,65,66,67,68,69,70,71]. In reported textile-based supercapacitors such materials like PPy [34,35,36,37,38], reduced graphene oxide (RGO) [56,57,58,59,60,61,62], graphene-based composites with PPy [63,71], PANI [64,65], carbon nanotubes (CNT) [66,67], MnO_2_ [68,69], and WO_3_ [70] particles were regarded as materials of supercapacitor active electrodes. To the fabrication of supercapacitor active electrodes simple dip-coating [34,35,36,37,38,56,57,58,59,60,63,64,65,66,68,69,70,71], as well as inkjet printing [61,62] and layer by layer methods [67] were used. The solid-state thread supercapacitors based on RGO exhibited the length specific capacitance of 18 μF/cm [56] and 110 mF/cm [57]. The reported gravimetric specific capacitance for the all-solid state fabric supercapacitors depended on material of active electrodes as well as textile fabric type. In case of PPy, RGO, RGO/PPy, and RGO/CNT composites-based fabric supercapacitors, the gravimetric specific capacitance were reported in the range of 103–325 F/g [34,35,37], 40–257 F/g [58,59,60,61,62], 114 F/g [71], and 200.4–2590 F/g [66,67], respectively.

In this paper, we report the formation of highly conductive PPy layers on polypropylene (PP) non-woven textile under low temperature (<0 °C) by using the reactive inkjet printing method with direct freezing of inks. This is a novel and entirely different approach from other methods of PPy deposition where the synthesis temperature is generally higher than 0 °C. In the proposed method, the textile substrate on which the PPy layer intend to form, is placed on a cooling plate of temperature ranged from 0 to −16 °C to ensure the direct freezing of inks droplets containing pyrrole and oxidant solution during the printing process. It allows to keep the frozen layers under the temperature below 0 °C until complete polymerization of pyrrole monomers. The obtained conducting fabrics were characterized by means of Raman spectroscopy and scanning electron microscopy (SEM). Thermal properties of inks used for PPy synthesis were tested with use of differential scanning calorimetry (DSC). The conducting properties (surface resistance) of the fabrics were measured by means of the two-probe method. Finally, an applicability of the produced PPy conductive fabrics was tested in symmetric all-solid-state supercapacitors.

## 2. Materials and Methods

### 2.1. Reagents and Materials

Pyrrole (98%), a product of SAFC (Wuxi, China), was supplied by Sigma–Aldrich (Saint Louis, MO, USA). Ammonium peroxydisulfate ((NH_4_)_2_S_2_O_8_, APS), analytical grade, and sulfuric acid (H_2_SO_4_, 98%) were supplied from Chempur (Piekary Śląskie, Poland). Poly(vinyl alcohol) (PVA), M_w_ = 72,000 g/mol was supplied from Poch (Gliwice, Poland). High purity (99.99%) electrolytically refined gold in a metallic form, was used in the process of vacuum deposition. The purity of gold was guaranteed by the supplier—Metals Mint Ltd. (Warsaw, Poland). All compounds were used as received. Polypropylene (PP) spun bonded non-woven fabric with the weight of 120 g/m^2^ and thickness of 0.52 mm was used as textile substrate. This type of fabric is commonly used in textile industry and its areal density can be from 60 to 200 g/m^2^. Depending on temperature, the reported conductivity of PP spun bonded non-woven fabric can be from 10^−12^ to 10^−14^ S/m [72].

### 2.2. Deposition of PPy Layers on Textile Substrates with Direct Inks Freezing

**Figure 1:**
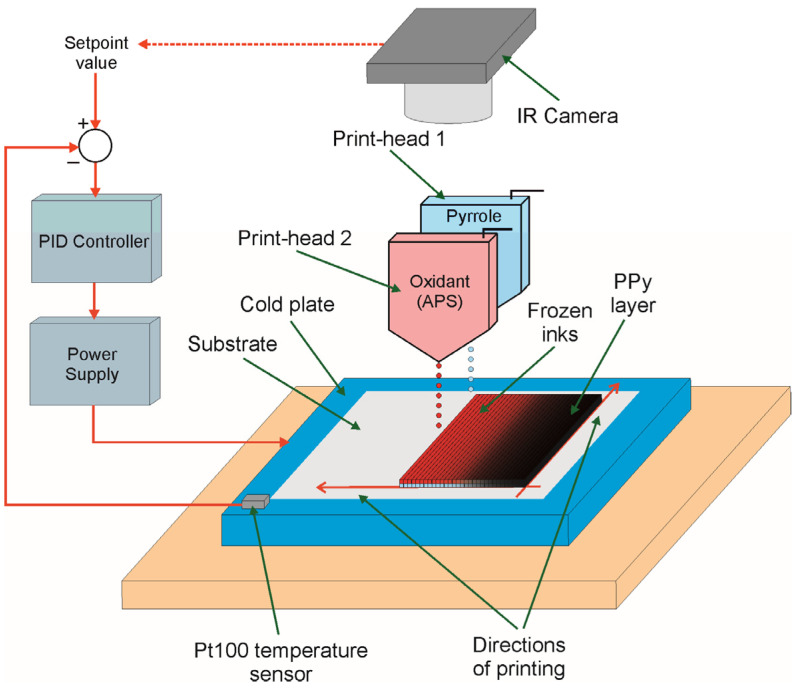
The idea of deposition of PPy layer with direct inks freezing.

The PPy electroconductive layers on the PP non-woven fabric were printed by the reactive inkjet printing technique based on direct inks freezing with the idea presented in Figure 1.

To realize this strategy, a prototype digital inkjet printer was used. It contained: (1) nanodispensing system, equipped with a two valve-jet print heads (ReaJet SK 1/080, Muehltal, Germany); (2) control system for generating a triggering impulses to the print-heads; (3) centralized ink pumping system; (4) two-axis positioning print heads motion system of a step resolution of 35 μm; (5) computer based control system with dedicated software to allow the selection of a printing pattern and control a printing process; (6) a cold plate represented by a Peltier cell of dimensions of 60 mm × 60 mm, where temperature was controlled by PID controller via voltage controlled power supply (Agilent E3644A, Santa Clara, CA, USA) on the base of setpoint value and current temperature measured by a miniature sensor Pt100 mounted to the surface of Peltier cell; and (7) a cooler represented by a dual tower cooler NH-D15 (Noctua, Wien, Austria) with heat-pipe layout and two fans. Prior to printing, Print-head 1 was filled by ink prepared as aqueous solution of pyrrole in the concentration of 0.89 M/dm^3^, while Print-head 2 was filled by ink prepared as aqueous solution of ammonium peroxydisulfate in the concentration of 0.89 M/dm^3^. The printed pattern was designed by using a vector graphic editor and then converted to a printer control data. On the basis of these data, the drive-electronics system was able to control the position of print-heads and releasing of drops. The PP non-woven fabric (substrate) was placed on a cold plate of temperature ranged from −16 °C to room temperature to ensure the direct freezing of inks droplets in case of printing in sub-zero temperatures and compared the properties of achieved layers with those prepared in above-zero temperatures. To eliminate an air spaces between cold plate and a back side of the PP non-woven fabric, a thin layer of water was put between them, which froze after cooling down the plate. The PPy layer was printed line by line onto the selected substrate in such a way that the first nozzle printed the selected line of pattern using an aqueous solution of pyrrole and then the second nozzle printed the same line of pattern using an aqueous solution of ammonium peroxydisulfate. The time of printing of the PPy layer of 1 cm^2^ by the prototype inkjet printer used in experiments was ca. 150 s. After finishing of printing the sample was further kept on a cold plate for 3 h to polymerize the pyrrole and then left at room temperature for 24 h for complete polymerization of pyrrole monomers. Although the temperature of cold plate was stabilized by using a PID control system, the temperature of front side of substrate was slightly higher due to thermal resistance of PP substrate and layer of frozen water placed between cold plate and substrate. Hence, to monitor the temperature of frozen inks the thermal camera ThermaCAM E55 (FLIR, Wilsonville, OR, USA) was used, which measured the temperature distribution on-line. On the base of these thermal images, a proper correction in the setpoint value of controller was introduced to achieve exact temperature for polymerization of pyrrole in a freezing state of inks. All experiments related to the PPy deposition process were realized in climatic room cooled down to the temperature of 15–16 °C in air atmosphere. In the case of the Peltier cell, which was used as a cold plate and the applied conditions, such as the cooling capacity of the cell and the possible minimum ambient temperature, the minimum temperature of cold plate that could be achieved was ca. −16 °C. Since reaching this temperature was difficult for a long time, it was decided to keep a certain margin during printing electrodes for supercapacitors and the minimum temperature to −12 °C was set.

### 2.3. Fabrication of Textile Solid-State Supercapacitor

**Figure 2:**
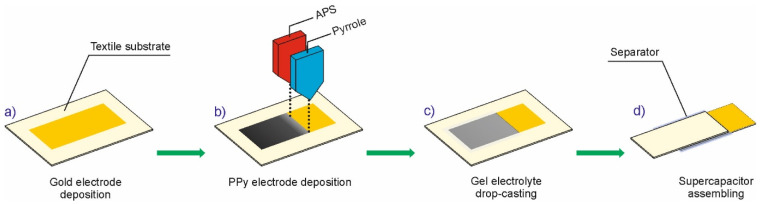
Detailed steps of fabrication of inkjet-printed textile supercapacitor.

The solid-state textile supercapacitor device was fabricated according to the scheme presented in Figure 2.

In the first step of supercapacitor fabrication, two rectangular Au electrodes od size of 10 mm × 20 mm were deposited on PP non-woven fabric with dimensions of 20 mm × 30 mm by using the Classic 250 Pfeiffer Vacuum system, Aßlar, Germany (Figure 2a). The resistance vapor source with evaporator current control was used. The deposition process was carried out for 5 min after obtaining a vacuum of 0.0002 Pa. In the second step, square shaped PPy layers, 10 mm in width and 10 mm in length, were printed on gold current collectors by using the method presented in Figure 1 (see also Figure 2b). During printing, the textile substrate was placed on a cold plate cooled then to desired temperature. After printing, the samples were kept at desired temperature for 3 h and then 24 h in room temperature. Next, two PP/Au/PPy electrodes were drop-casted with the H_2_SO_4_/PVA gel electrolyte and then picked out for air drying at room temperature for 24 h. The H_2_SO_4_/PVA gel electrolyte was prepared as follows: 1 g of H_2_SO_4_ was added into 10 mL of deionized water and heated to 85 °C under stirring. After the mixture reached a temperature of 85 °C, 1 g of PVA was added gradually. The whole mixture was kept under heating and stirring until the solution became clear. In order to assemble the solid-state sandwiched supercapacitor device, the H_2_SO_4_/PVA gel electrolyte coated two PP/Au/PPy electrodes (Figure 2c) were separated by a glass microfiber filter (Grade GF/F, Whatman, Maidstone, UK) and subsequently pressed together under a pressure of ~1 MPa for 10 min to make a quasi-solid state supercapacitor assembly (Figure 2d).

### 2.4. Characterization

#### 2.4.1. Scanning Electron Microscopy

To determine PPy morphology and to assess the distribution of PPy on the textile surfaces the scanning electron microscopy (SEM) was used. For this purpose, a scanning electron microscope with field emission TESCAN VEGA3–EasyProbe equipped with VEGA TG software with high vacuum mode (SE) option; accelerating voltage 7 kV) (TESCAN Brno, s.r.o., Brno, Czech Republic) was used. The samples were not coated before the measurements.

#### 2.4.2. Raman Spectroscopy

Raman spectra were acquired using a RamanScope III FT-Raman spectrometer (Bruker, Billerica, MA, USA). As a source of excitation light, the Nd:YAG laser line λ = 1065 nm was used, and a nominal laser light power was 5 mW. The spectral resolution was 4 cm^−1^, and 512 scans were averaged to obtain high quality spectra. The polynomial baseline was extracted from each spectrum and next the spectra were slightly smooth with use of Savitzky–Golay method. Finally, the spectra were normalized to the total integral intensity.

#### 2.4.3. Surface Resistance

**Figure 3:**
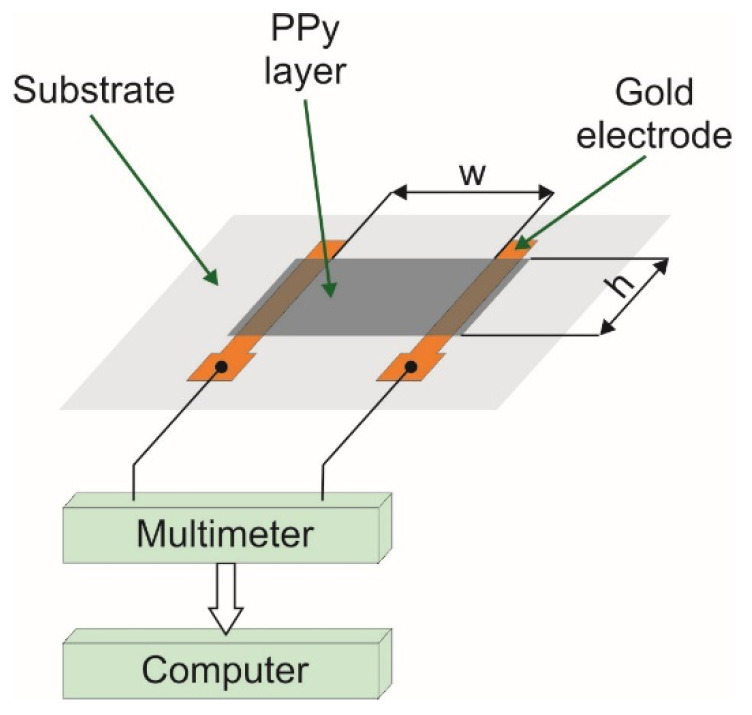
Two-point probe setup to measure the resistance of PPy layer.

The surface resistance of the printed samples was determined according to the American Association of Textile Chemists and Colorists Test Method 76-2005 using the two-point probe method [73]. Prior to inkjet printing of PPy layers, two strips of gold (2 mm width and 10 mm length) were deposited on PP non-woven fabric with the distance of 6 mm using the Classic 250 Pfeiffer Vacuum system (Figure 3). The resistance vapor source with evaporator current control was used. The deposition process was carried out for 5 min after obtaining a vacuum of 0.0002 Pa. Next, two copper wires were glued to the ends of the gold electrodes by using the Amepox ELPOX AX 15S (Amepox, Lodz, Poland) electrically conductive silver epoxy resin and finally the PPy layer 12 mm with and 6 mm height was deposited on top of electrodes by using the method presented in Figure 1. The curing temperature of the silver epoxy resin was 130 °C. According to the adopted method, the surface resistance *R_s_* given in Ω/sq. was calculated from equation (1) on the base of bulk resistance of PPy layer measured by multimeter Picotest M3500A (Picotest, Phoenix, AZ, USA) and size of the layer between gold electrodes (Figure 3).

(1) $$R_{s}=\frac{h}{w}R$$

where *R* is the bulk resistance of PPy layer measured by multimeter, *w* is the distance between inner edges of gold electrodes, and *h* is the height of PPy layer. All the bulk resistance measurements we realized under standard conditions for textile testing according to ISO 139 reference. According to this reference, the temperature and humidity in lab space were kept as 20 °C and 65%, respectively.

#### 2.4.4. Supercapacitor Characterization

All the electrochemical measurements were performed using Interface 1010E (Gamry Instruments, Warminster, PA, USA) electrochemical workstation. Before testing, each supercapacitor device was activated by cyclic voltammetry (CV) using potential window of −0.5 V to 0.5 V for 20 cycles. The electrochemical impedance spectroscopy (EIS) measurements were performed at open circuit potential with a sinusoidal signal over a frequency range from 0.01 Hz to 1 MHz at an amplitude of 5 mV. The specific capacitance derived from the galvanostatic charge/discharge (GCD) curves was calculated based on the following formula [74], which takes into account a non-linear profile of the supercapacitor discharge curve:

(2) $$C_{S}=2\cdot I_{dis}\frac{\int U\left(t\right)\cdot dt}{\varepsilon \cdot \left(U_{max}^{2}-U_{min}^{2}\right)}$$

where: *I_dis_* is the current (A) applied to the device, *ε* is the superficial area (cm^2^) of electrodes or mass (g) of active electrodes for the areal or gravimetric specific capacitance, respectively, *U*(*t*) is the discharge curve of the tested supercapacitor, *U_max_* is the starting voltage of the discharge after the drop due to the equivalent distributed resistance (V), *U_min_* is the final voltage at the end of the discharge (V), *t* is the discharge time (s), and IR represents the voltage drop appearing at the start of the discharge process, caused by the internal resistance of the device. The energy density (*E_D_*) as well as power density (*P_D_*) of the device were calculated using the following formulas [75]:

(3) $$E_{D}=\frac{0.5\cdot C_{S}\cdot V^{2}}{3.6}\;,\;Wh/kg$$

(4) $$P_{D}=\frac{E_{D}\cdot 3600}{t}\;,\;W/kg$$

where: *C_s_* (F/g) is a supercapacitor specific capacitance and *V* is the voltage change during the discharge process after IR drop.

#### 2.4.5. Differential Scanning Calorimetry (DSC)

Thermograms for all investigated samples were collected with the Differential Scanning Calorimeter DSC 3 (Mettler Toledo, Columbus, OH, USA). The temperature and heat flow were calibrated using indium and zinc melting point standards. DSC curves were obtained at a scan rate of 2 °C/min in a temperature range −50 °C to 20 °C in ambient atmosphere. All materials were placed in sealed aluminum pans just before measurements. Samples were firstly cooled down from room temperature (20 °C) to −50 °C and immediately heated up again to room temperature. Two scans were done, and only the results of the second heating run were analyzed.

## 3. Results

### 3.1. Surface Morphology of Printed PPy Layers

**Figure 4:**
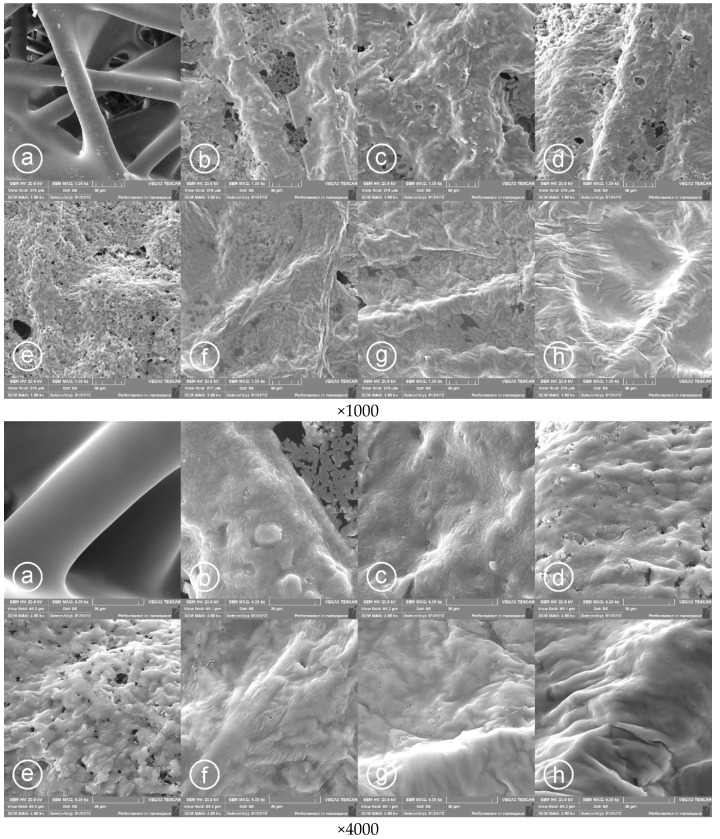
SEM of PPy/PP composite with different polymerization temperature of PPy layers during printing taken at magnifications of ×1000 and ×4000 and: (**a**) PP fabric, (**b**) 23 °C, (**c**) 10 °C, (**d**) 2 °C, (**e**) −3 °C, (**f**) −6 °C, (**g**) −9 °C, and (**h**) −12 °C.

The SEM images of the PPy/PP electrodes were taken with magnifications of ×1000 and ×4000 for the samples prepared at different polymerization temperature of PPy. As Figure 4 shows, the PPy layers were successfully inkjet-printed on the PP non-woven fabric. The PPy layers printed under temperature range from 23 °C to 0 °C had irregular and non-smooth structure (Figure 4b–d). In contrast, if the polymerization temperature dropped down below 0 °C the PPy layers appeared to be more homogeneous and smoother (Figure 4e–h). In particular, PPy layers prepared at −12 °C showed fully covered and continuous layered structural morphology (Figure 4h). This result suggests that the polymerization temperature had a great impact on morphological structure of PPy layers on PP fabric. The result is consistent with the previous reported work [49]. The films of PPy formed at sub-zero temperatures were smooth, coherent, and mechanically stronger compared to those prepared at higher temperatures. This may be due to the less aggregation of PPy particles during nucleation process under low temperature. The mode of nucleation at the initial stage of the formation of nanoparticles plays an important role in determining the shape of the resulting particles as well as their aggregation behavior [76]. Heterogeneous nucleation over pre-formed particles can cause irreversible aggregation of nanoparticles during the synthetic process. Analyzing the obtained results presented in Figure 4, it seems that lowering the polymerization temperature can limit the heterogeneous nucleation over the pre-formed particles and reduces the aggregation of PPy particles, which results in a more uniform and smoother film on the PP fabric. This probably due to the water crystallization leading to micro phase-separation. In result of ice crystals formation at sub-zero temperatures, the PPy synthesis occurs in condensed and confined space. Such conditions prevent against the aggregation of PPy particles. Since the PPy films obtained at temperatures below −6 °C are fully homogeneous and smooth (Figure 4f–h).

### 3.2. DSC Analysis

**Figure 5:**
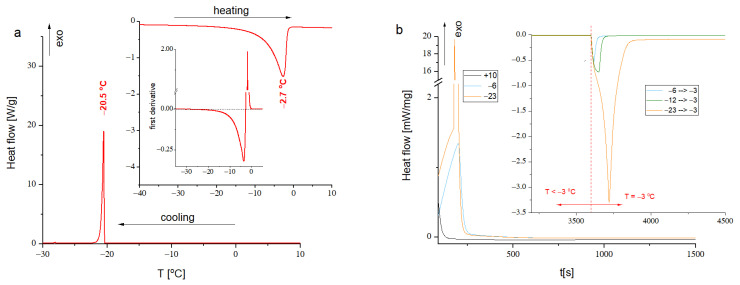
(**a**) DSC scan of aqueous solution of Py/APS equimolar mixture—cooling ramp and heating ramp shown in inset, additionally the first derivative of heating ramp for Py/APS mixture is shown; (**b**) results of isothermal measurements for the Py/APS equimolar mixture performed at various temperatures, as inset the isothermal measurements relating to melting process triggered by the temperature jump from low temperature to −3 °C (the temperature jump was marked by dashed line).

To compare the physical state of reaction mixtures at different temperatures the DSC measurements were performed. Figure 5a presents the dynamic DSC scans of Py/APS equimolar mixture. The water freezing point was found at −20.5 °C. It is important to notice that the water freezing point equal to 273 K (0 °C) is characteristic only for pure water. Due to cryoscopic effect it is shifted in the solutions and gels toward lower temperatures [77,78]. Moreover, the differential scanning calorimetry is a dynamic method, so the transition points are influenced by the heating and cooling rates. The transition temperatures determined at the heating/cooling rate (10 °C/min) used in these studies are quite far from equilibrium phase transition temperature and water could exist in supercooled state. Hence, it seems that the most important is to look at the heating scans. The endothermic peak relating to water melting is very broad and asymmetric, what means that the water melting in investigated systems is not clearly the first order transition. The main water melting (the fastest at 2.7 °C) is preceded by the pre-melting process started at −30 °C. This is quite commonly observed phenomenon in polymer-water systems [79,80,81,82,83]. This is a crucial observation for the PPy synthesis by the reactive inject printing method at low temperatures. The DSC results proved that the reaction mixture at the temperature range used for PPy layer synthesis (from 0 to −16 °C) is heterogenous system containing ice crystals and confined between them concentrated solution of growing PPy chains, and reactants Py and APS. Such conditions should favor formation of percolation pathway and in result designation of the material with higher conductivity in comparison to the PPy layer obtained at above-zero temperatures. The isothermal measurements performed for equimolar Py/APS reactive mixture at some selected temperatures (Figure 5b) showed that both water crystallization and melting are extended in time and finished after ca. 10–15 min. For comparison, the isothermal DSC experiment was also performed at +10 °C. Obtained thermogram did not show any peak, what proved that observed effects at sub-zero temperatures corresponded to water phase transitions. Sample isothermally measured at sub-zero temperatures after one hour were heated up to −3 °C—see inset to Figure 5. The slow pre-melting process started, and the endothermic melting peak area is the higher, the lower the freezing temperature was applied. The DSC results evidently showed that the PPy formation during the low temperature reactive inject printing occurs in the heterogenous environment containing ice crystals and more concentrated (in comparison to initial solutions) reactive mixture.

### 3.3. Surface Resistance of Printed PPy Layers

**Figure 6:**
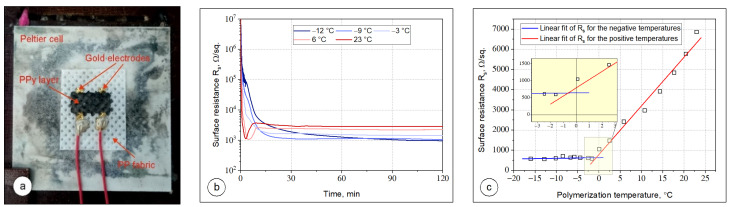
Surface resistance measurements of printed PPy layers: (**a**) printed samples placed on a cold plate during PPy layer deposition and polymerization; (**b**) time changes in the PPy layer resistance during deposition and polymerization processes for the polymerization temperature of −12, −9, −3, 6, and 23 °C; and (**c**) final surface resistance of PPy layers vs. polymerization temperature.

The surface resistance of the PPy layers printed on PP fabric against the polymerization temperature was analyzed in a transient and steady states conditions. To analyze the effect of polymerization temperature on the surface resistance of PPy layers in transient state, firstly, two gold electrodes were deposited on PP fabric by physical vapor deposition (PVD) method. Then the sample was placed on a cold plate (Peltier cell) and a rectangular PPy pattern of 6 mm × 12 mm was printed between gold electrodes (Figure 6a). The resistance of PPy printed layer between electrodes was measured on-line by a multimeter Picotest M3500A for 120 min after printing. The temperature of the cold plate was varied from −12 °C to 23 °C.

Figure 6b presents a series of surface resistance transients obtained during the formation of PPy layer on the PP fabric at different polymerization temperatures −12, −9, −3, 6, and 23 °C. The two types of curves can be distinguished. Curves recorded at sub-zero temperatures exhibit two regions—first relating to a fast decrease in surface resistance and second representing a gradual decrease of surface resistivity. For samples prepared above the water freezing point the transient characteristics are more complex. Firstly, very fast decrease of surface resistivity is observed resulting in PPy formation. After 4–6 min of deposition the minimum of the surface resistivity on transient characteristics is visible (the process temperature is higher, the earlier the minimum occurs). Observed trends can be explained basing on the nucleation and growth of PPy particles under different temperature regimes. Even as the drops of the aqueous solution of pyrrole and APS are released from the nozzle head onto the PP substrate, they instantaneously initiate the nucleation, growing the PPy particles. As the time increases, the concentration and size of PPy particles suspended in a mix of aqueous solutions of pyrrole and APS increases, resulting in a rapid decrease in surface resistance. As can be seen in Figure 6b, the time of nucleation and growth of PPy particles strongly depends on polymerization temperature. For the temperatures 6 and 23 °C, when the mixture of aqueous solution of pyrrole and APS is not frozen, the process of nucleation and growth of PPy particles is faster and after 4–5 min the surface resistance of printed layer achieves a minimum value of surface resistance. At sub-zero temperatures, the mixture of aqueous solution of pyrrole and APS is partially frozen and the nucleation process and the growth of PPy particles significantly slows down due to the confined space. It can be noticed that as the polymerization temperature lowers, the minimum of final surface resistance was achieved. If the polymerization temperature was −12 °C the minimum value of surface resistance after more than 90 min was achieved.

**Figure 7:**
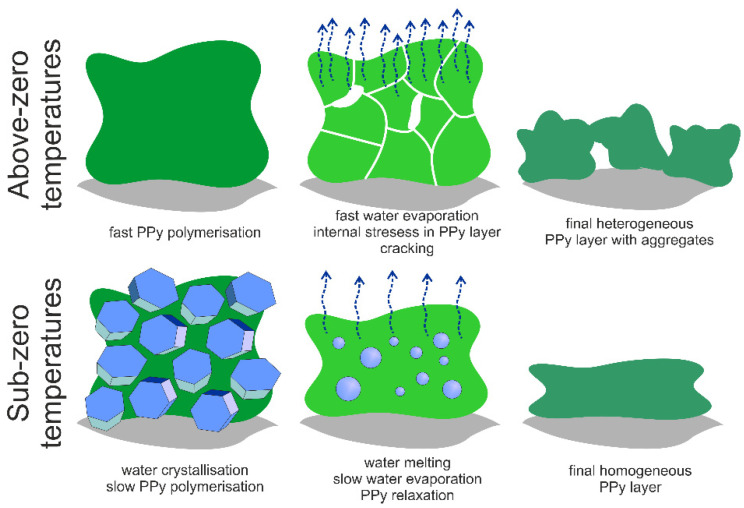
Schematic representation of the processes leading to different morphology of PPy layer printed on the textile (gray areas correspond to textile support, blue polyhedrons represent ice crystals, blue circles represent melting ice crystals, and water evaporation is marked by arrows).

It is important to notice that if the PPy polymerization run in solution (at high temperatures) the resistivity starts to slightly increase again after 4–6 min of polymerization. This effect probably due to water evaporation, leading to fast PPy aggregation and discontinuity of the layer of reactive mixture. In result, some pathways for charge transport break and the final morphology of PPy layer is strongly inhomogeneous. This effect is not observed at sub-zero temperatures, where the transient characteristics were found monotonous. The polymerization process slows down with temperature decrease, but low temperature protects the growing layer against fast water evaporation and cracking of the final PPy layer. The ice melting, and next water evaporation after the polymerization process does not strongly induce the continuity of the polymer conductive layer as PPy is swellable in water what ensures slow water removing and in consequence sufficient time for layer relaxation [84,85,86]. The proposed mechanisms of the formation of PPy layer onto the textile support at various temperature regimes are proposed in Figure 7.

After solvent evaporation from the samples at room temperature for 24 h, the surface resistance of the printed layers was measured at a steady state. The determined values for PPy layers deposited at various temperatures are shown in Figure 6c. The obtained results were fitted by linear functions in two regions separately (below and above 0 °C). The slopes in the changes of surface resistance of printed PPy layer significantly differ in these regions. For the samples obtained at temperatures varied between 23 and 0 °C, the surface resistance linearly decreases from 6335 Ω/sq. to 792 Ω/sq. A large change in the surface resistance result from the scale of defects in the PPy layer formed at different polymerization temperatures (see Figure 4b–d). The highest surface resistance of PPy layers was noticed if the polymerization temperature was 23 °C. At this temperature the irregular PPy structure with cracks and holes which restrict the flow of electrical current in the bulk is observed. The number of defects/holes gradually decreases when the polymerization temperature decreases, which leads to the lower surface resistance of PPy layers. For the sub-zero temperatures, the surface resistance further linearly decreases, however with a very small slope of 3.4 Ω/sq. per 1 °C. The lowest surface resistance of 584 Ω/sq. was measured when the polymerization temperature was −16 °C. It can be attributed to the possible limitation of heterogeneous nucleation over the pre-formed particles and reducing the aggregation of PPy particles at low temperatures, which results in a more uniform and smoother film of PPy on the PP fabric (see Figure 4e,f). Two regression lines intersect at ca. −0.8 °C (see inset in Figure 6c). This is probably the threshold temperature between the solid and liquid phases of the mixture of aqueous solutions of pyrrole and APS. This result is comparable to this found by calorimetric dynamic measurements where the melting peak of equimolar solution of Py and APS was found at −2.7 °C. It proves that partially frozen environment prevents against the defect formation in the PPy layer, thereby the surface resistance of PPy layer does not change significantly, whereas if the PPy polymerization occurs in the liquid state, defects are induced as a result of the aggregation of PPy particles during their nucleation and growth and fast water evaporation. Thus, we can conclude that an increase in the polymerization temperature affects the number of defects in the PPy layer and a significant increase in its surface resistance.

### 3.4. Raman Spectroscopy of Printed PPy Layers

Generally, the conductivity of conjugated polymers strongly depends on their reduction/oxidation states which determine number of free charge carriers. This is one of the most important, and initially expected effects which could explain the differences in electrical conductivity of PPy layers formed at different temperatures. The oxidation state of PPy depends on the synthesis method used for polymer preparation, pH, electrical field application, and presence of oxidants or reductants in the system. Thus, to confirm the model proposed to explain differences in surface conductivity of PPy layer imprinted onto the textile support (see Figure 7) it was necessary to verify the chemical structure and degree of oxidation of obtained PPy layers at different temperatures.

**Figure 8:**
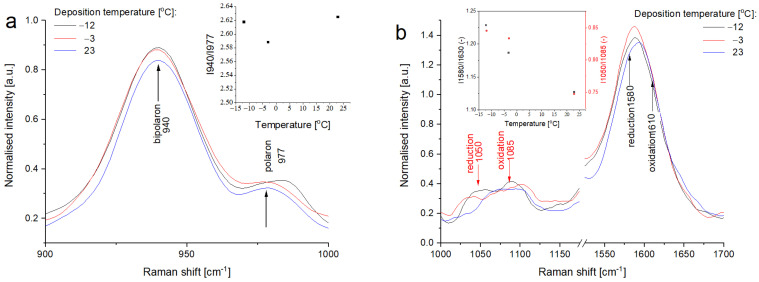
Normalized Raman spectra of some selected PPy layers printed on the textile at different temperatures in the region: (**a**) sensitive to the polaron/bipolaron ratio and (**b**) sensitive to the oxidized/reduced state.

Vibrational spectroscopy methods are very useful to characterize the oxidation/reduction degree of conducting polymers including polypyrrole. Surprisingly, the Raman spectra recorded for all tested samples are practically identical—see Figure 8. The ratio between the intensities of lines at 940 and 977 cm^−1^ sensitive to polar/bipolaron presence is practically independent of the temperature od PPy synthesis [87]. In the regions sensitive to oxidation/reduction some very small differences are observed. Slight increase in intensities of lines 1050 and 1580 cm^−1^ with decreasing temperature suggests that the reactive printing at low temperatures results in a little bit more reduced PPy [87]. However, the observed differences are so small, that evidently, they could not be responsible for the differences in surface resistivity found for PPy layer deposited at various temperatures. Thus, presented results of Raman spectroscopy excluded the chemical aspects as a reason of differences in sample conductivity.

### 3.5. Electrochemical Performance of PPy Based Textile Solid-State Supercapacitors 

In assembled textile solid-state supercapacitors PPy material electrodes were deposited on PP/Au interface. The layer of Au vacuum deposited on PP substrate was a current collector. The surface resistance of this interface measured by using 4-point probe method was 13.4 ± 2.4 Ω/sq., which was much lower than the surface resistance of PPy layer.

**Figure 9:**
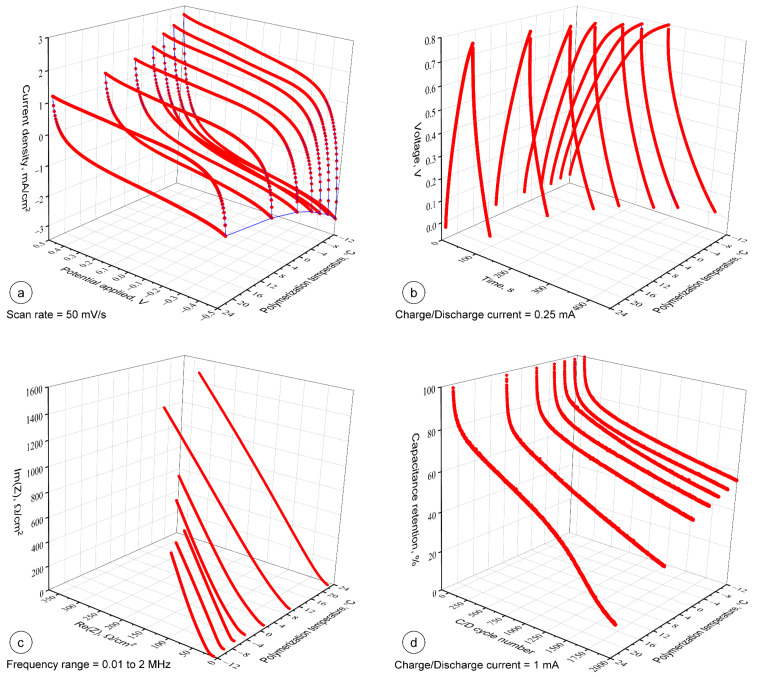
Electrochemical properties of PPy based textile supercapacitors vs. polymerization temperature (**a**) CV curves measured at the same scan rates, (**b**) constant current charge/discharge curves, (**c**) EIS spectra of the supercapacitor devices, and (**d**) capacitance retention of the supercapacitor devices as a function of charge/discharge cycle number.

Electrochemical properties of the assembled PPy based textile solid-state supercapacitors with respect of the PPy polymerization temperature is recorded, as shown in Figure 9. Figure 9a presents the CV curves of the supercapacitors at the scan rate of 50 mV/s in a potential window of −0.5–0.5 V. All the supercapacitor devices showed the quasi-rectangular shape of CV irrespective of the polymerization temperature of PPy, accompanied by the mechanism of double layer capacitive energy storage coupled with diffusion controlled redox exchange behavior of the cells [88]. The supercapacitors assembled with PPy prepared at sub-zero temperatures showed slightly larger charge propagation in the CV curves compared to those containing PPy prepared at above-zero temperatures. This effect can be attributed to the higher electrical conductivity and homogeneous morphological structure of the PPy layers prepared at low temperature (see Figure 4), qualitatively indicating the higher capacitance. As shown in Figure 9b, the GCD curves at the current density of 0.25 mA/cm^2^ for all supercapacitor devices reparent the quasi-triangular shape and the charging/discharging time increases with the decreasing PPy polymerization temperature. This is in agreement with the results obtained from the CV test. At the beginning of discharging curve, there is a small voltage drop that arises from the ohmic loss due to the internal resistance of the device. The average internal resistances calculated from the voltage drops are 74.9 and 94.7 Ω for the PPy layers formed at sub-zero and above-zero temperatures, respectively. The internal resistances are quite low considering the solid-state design of the textile supercapacitors.

In Figure 9c, Nyquist plots of supercapacitors with PPy electrodes synthetized at different temperatures are compared. There was no visible semicircle observed in the high frequency region, suggesting a low charge transfer resistance. This implies a favorable ionic transport across the solid phase of PPy layers over textile substrate at the interface with gel electrolyte. The equivalent series resistances of the devices were measured between the range of 7–11 Ω. This suggests a low contact resistance at the interface of the active material and substrate. It should be noted that no influence of the PPy polymerization temperature on the equivalent series resistance of the devices was observed. In the low frequency region, the nearly vertical line indicates a low resistance for the diffusion of ions and good capacitive behavior of the devices. The cycling stability of the supercapacitors is important for practical applications. To evaluate it, we conducted solid-state supercapacitors, for 2000 sequential galvanostatic charge/discharge cycles at a constant current of 1 mA/cm^2^. Figure 9d shows the variation in the capacitance retention of the tested supercapacitors as a function of the charge/discharge cycle numbers and PPy polymerization temperature. As can be seen at the beginning the capacitance of the device rapidly drops to 80% irrespective of PPy polymerization temperature and then gradually start attaining a steady state curve. However, it was observed that the device made of PPy polymerized at low temperatures showed less decrease in capacitance than the devices assembled with PPy printed above 0 °C. The highest value of capacitance retention after 2000 cycles of 55.4% was attained for the PPy layer prepared at −12 °C, while 14.8% capacitance retention was calculated for the PPy prepared at 23 °C. It can be noticed that the presented textile supercapacitors especially with PPy material electrodes polymerized at sub-zero temperatures exhibited much higher cycling stability than PPy coated nylon Lycra fabric electrode (~12.5% of the initial capacitance after 500 charge/discharge cycles) [34], PPy coated knitted fabric electrode with 90% cotton and 10% Lycra (50% of capacitance retention after 500 cycles) [35], cotton electrodes coated with undoped PPy nanorods (59% of capacitance retention after 500 cycles) [37], and supercapacitor based on cotton yarn coated with PPy nanotubes (56.7% of capacitance retention after 700 cycles) [38]. In comparison, the presented textile supercapacitor with PPy material electrodes polymerized in the temperature of −12 °C exhibited a 70.8% and 68.1% of capacitance retention after 500 and 700 cycles, respectively.

**Figure 10:**
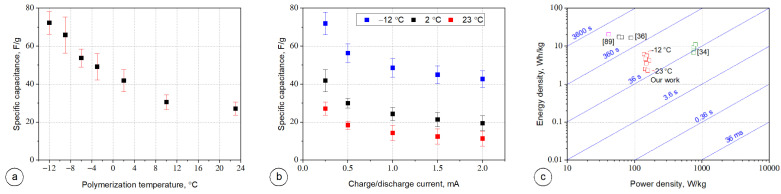
Electrochemical performance of Ppy based textile supercapacitors: (**a**) specific capacitance vs. polymerization temperature for current charge/discharge equal to 0.25 mA, (**b**) specific capacitance vs. current charge/discharge for different Ppy polymerization temperature, and (**c**) Ragone plot of the specific energy vs. specific power.

The specific capacitance values of the supercapacitor devices constructed with PPy prepared at different polymerization temperatures were calculated according to the discharge curves and shown in Figure 10a,b. As Figure 10a shows the specific capacitance increases if the PPy polymerization temperature decreases. It can be attributed to the changes in the morphology of PPy layers during polymerization under varied temperatures. As described in Figure 4 and Figure 7, the PPy layers prepared at low temperature showed more uniform and smoother surfaces and cover entire surface area of the fabric. This facilitates the formation of a continuous conductive active area of the electrode and hence improved capacitance. The highest and the lowest values of capacitance of 72.3 and 27.1 F/g (at 0.6 A/g) were obtained for the supercapacitors with PPy prepared at −12 and 23 °C, respectively and compared with some lately reported values obtained for the PPy based double layer textile supercapacitors, as shown in Table 1.

Figure 10b shows the specific capacitance of PPy-based supercapacitors obtained at different charge/discharge current for PPy electrodes polymerized at temperatures of −12, 2, and 23 °C. For each tested supercapacitor the specific capacitance decreased as the charge/discharge current increased. This decreasing of capacitance at higher current rates is due to less ionic penetration in the PPy material electrodes compared to the lower current rate, which is a typical behavior of electrochemical supercapacitors.

Figure 10c presents the comparison of the energy and power densities between our results and previous reported work on PPy-based textile supercapacitors [34,36,89]. The power density obtained for the as-prepared PPy-based supercapacitors was independent of the PPy polymerization temperature and it retained ca. 150 W/kg, while the energy density increased as the PPy polymerization temperature decreased. The smallest and the highest values of energy density of 2.30 and 6.12 Wh/kg were obtained for the polymerization temperature of 23 and −12 °C, respectively. The devices constructed within this work indicated reasonably good energy and power densities compare to the previously reported textile supercapacitors [34,36,89]. The diagonal time-lines visible in Figure 10c are representative for the so-called “characteristic times” and they reflect the device running time at the specified power [90]. Comparing the operating times of supercapacitors assembled with PPy-based electrodes printed at different temperatures with the reported operating times of PPy-based textile supercapacitors, the results were varied, mainly due to the different charge/discharge current applied during the tests, used doping of PPy like naphthalene-2,6-disulfonic acid disodium salt (Na_2_NDS) [34], p-toluenesulfonic acid (p-TS) [35], and lignosulfonate [89].

## 4. Conclusions

Performed study has demonstrated an application of the reactive inkjet printing to fabricate PPy layers on textile substrates with direct freezing of inks. The PPy was synthetized in situ by oxidative polymerization of pyrrole using APS in partially frozen state. SEM and DSC analysis and Raman spectroscopy as well as a decrease in surface resistance revealed that PPy was successfully deposited on PP textile substrates at sub-zero temperatures. It has been shown that the water crystallization strongly influences the process of in situ formation of PPy layer on textile, resulting in smoother morphology of PPy layer and its higher electrical conductivity. The model assuming water crystals formation at sub-zero temperatures has been proposed to explain observed phenomena and discussed. By comparing the electrochemical performance of the as-assembled textile supercapacitors with PPy material electrodes printed at different temperatures, it could be concluded that the PPy polymerized at sub-zero temperatures exhibited better supercapacitive performance. It can be attributed to the homogeneous and continuous layered morphological structure of PPy under low polymerization temperature. The conducted analyses proved that supercapacitor with PPy material electrodes printed under the temperature of −12 °C exhibited the highest specific capacitance of 72.3 F/g at a current density of 0.6 A/g and delivered an energy density of 6.12 Wh/kg while maintaining the power density of 139 W/kg. Additionally, the supercapacitor shows a good electrochemical stability compared to the previously reported results [34,35,37,38] and retained 55.4% of its initial capacitance after 2000 cycles. This work demonstrated a promising future of the reactive inkjet printing technique with direct freezing of inks for a large-scale fabrication of PPy-based conductive smart textiles especially for disposable power devices.

## Figures and Tables

**Table 1:** Comparison of performance of PPy based double layer textile supercapacitors.

Textile Substrate	Electrode Material	PPy Deposition Method	Capacitance, F/g	Capacitance, mF/cm^2^	Reference
Knitted fabric (80% Nylon and 20% Lycra)	PPy doped with Na_2_NDS	Dip-coating	108.5 (1 A/g)	not given	[34]
Knitted cotton fabric	PPy doped with NaSSA	Dip-coating	not given	1167.9 (1 mA/cm^2^)	[36]
Cotton fabric	PPy nanorods	Dip-coating	325 (0.6 mA/cm^2^)	not given	[37]
Knitted cotton fabric	PPy doped with 5-sulfosalicylic acid	Dip-coating	not given	450 (1 mA/cm^2^)	[38]
PP non-woven fabric	PPy	Reactive inkjet printing in sub-zero temperature	72.3 (0.6 A/g)	30.5 (0.25 mA/cm^2^)	This work

## Data Availability

The data presented in this study are available in the databases of the authors at the Lodz University of Technology.
